# Fluconazole Is Neuroprotective via Interactions with the IGF-1 Receptor

**DOI:** 10.1007/s13311-022-01265-0

**Published:** 2022-07-13

**Authors:** Valerie Toodle, Myoung-Hwa Lee, Muzna Bachani, April Ruffin, Sneha Vivekanandhan, Nasir Malik, Tongguang Wang, Tory P. Johnson, Avindra Nath, Joseph P. Steiner

**Affiliations:** 1 Section of Infections of the Nervous System, National Institute of Neurological Disorders and Stroke, National Institutes of Health, Room 7C-103; Bldg. 10, 10 Center Drive, Bethesda, MD 20892 USA; 2 Translational Neuroscience Center, National Institute of Neurological Disorders and Stroke, National Institutes of Health, Room 7C-105; Bldg. 10, 10 Center Drive, Bethesda, MD 20892 USA

**Keywords:** Neurodegeneration, Neuroprotection, Fluconazole, Neurotherapeutics, Insulin growth factor-1 receptor

## Abstract

There is a continuing unmet medical need to develop neuroprotective strategies to treat neurodegenerative disorders. To address this need, we screened over 2000 compounds for potential neuroprotective activity in a model of oxidative stress and found that numerous antifungal agents were neuroprotective. Of the identified compounds, fluconazole was further characterized. Fluconazole was able to prevent neurite retraction and cell death in in vitro and in vivo models of toxicity. Fluconazole protected neurons in a concentration-dependent manner and exhibited efficacy against several toxic agents, including 3-nitropropionic acid, N-methyl D-aspartate, 6-hydroxydopamine, and the HIV proteins Tat and gp120. In vivo studies indicated that systemically administered fluconazole was neuroprotective in animals treated with 3-nitropropionic acid and prevented gp120-mediated neuronal loss. In addition to neuroprotection, fluconazole also induced proliferation of neural progenitor cells in vitro and in vivo. Fluconazole mediates these effects through upregulation and signaling via the insulin growth factor-1 receptor which results in decreased cAMP production and increased phosphorylation of Akt. Blockade of the insulin growth factor-1 receptor signaling with the selective inhibitor AG1024 abrogated the effects of fluconazole. Our studies suggest that fluconazole may be an attractive candidate for treatment of neurodegenerative diseases due to its protective properties against several categories of neuronal insults and its ability to spur neural progenitor cell proliferation.

**Supplementary Information:**

The online version contains supplementary material available at 10.1007/s13311-022-01265-0.

## Introduction

Neurological disorders are the leading cause of disability and the second leading cause of death worldwide [1]. Furthermore, as the global human population ages, non-communicable neurological diseases, such as neurodegenerative diseases and motor neuron diseases, are predicted to become an even larger burden [2, 3]. Examples of these diseases include Alzheimer’s disease, Parkinson’s disease, and amyotrophic lateral sclerosis (ALS). While each of these neurodegenerative diseases is clinically and pathologically distinct, some common pathophysiological mechanisms underlying neuronal injury exist. Excitotoxicity, oxidative stress, synaptic pruning, retraction of dendrites, mitochondrial dysfunction, and activation of caspase pathways have all been implicated in neurodegeneration [4]. However, effective therapeutic targeting of neurodegenerative disease processes remains very challenging.

Currently, therapies for neurodegenerative diseases are aimed at symptom management and therapies targeted at excitotoxicity or oxidative stress have had a minimal effect on disease course [5]. Recently, the FDA approved aducanumab for treatment of Alzheimer’s disease [5, 6], which reduces amyloid plaques. However, the clinical benefits of this drug remain unknown. Furthermore, a recent analysis of 113 randomized placebo-controlled trials examining non-FDA-approved potential disease modifying neurodegenerative therapies showed that there was no benefit to patients from any of the proposed interventions [7]. Although great strides have been made in understanding the pathogenesis of neurodegenerative diseases, no cures or preventions exist. As the burden of neurodegenerative diseases continues to grow, there is an urgent need to develop interventions which can prevent neuronal loss and thereby halt or significantly slow the progression of these diseases.

An ideal drug candidate would be broadly neuroprotective, and therefore useful across a spectrum of neurodegenerative diseases, as well as being safe and tolerable for long-term use. This is of key importance as preventative strategies will need to be initiated prior to neuronal loss and onset of symptoms. Symptomatic patients may already have irreversible neuronal damage, reducing the potential of identifying compounds that may be protective during neurodegenerative processes. Unfortunately, developing new neuroprotective compounds requires a substantial investment of time and resources and carries a high risk for failure. However, repurposing approved drugs for alternative uses has proven beneficial for several diseases, including cancer and infectious diseases, and reduces both the fiscal investment and the time it takes for these drugs to ultimately benefit patients (reviewed in [8]). Importantly, these compounds also have long-term safety data associated with them and thereby could be used in clinical trials for intervention of disease in patients prior to symptom onset. For these reasons, we screened a panel of FDA-approved drugs and compounds in an in vitro neurotoxicity assay to assess their potential repurposing as neuroprotective agents.

In this study, we examined the neuroprotective potential of a panel of drugs and compounds and found that the imidazole class of drugs displayed neuroprotective properties. In particular, fluconazole showed broad protection against several different mechanisms of neurotoxicity both in vitro and in vivo. Furthermore, we demonstrate that fluconazole was able to prevent neuronal death, protect against neurite degeneration, and induce neurogenesis. Fluconazole altered neuronal signaling by increasing expression of the insulin-like growth factor 1 receptor, decreasing cyclic adenosine monophosphate (cAMP) levels, and increasing Akt signaling. These data suggest that fluconazole, an FDA-approved drug, may be a worthy candidate for further investigation as a neuroprotective compound.

## Materials and Methods

### Ethical Approval

All human tissue samples were collected after approval by the institutional review board in the Office of Human Subjects Research and Protection at the NIH. All murine tissues were collected according to the recommendations in the Guide for the Care and Use of Laboratory Animals of the NIH and studies were approved by the Johns Hopkins Medicine Institutional Animal Care and Use Committee (permit number: MO02M269).

### Reagents

All media, sera, and supplements were obtained from Thermo Fisher Scientific (Grand Island, NY) unless otherwise noted. All primary antibodies and LY294002 were obtained from Cell Signaling Technology ([CST], Beverly, MA). Fluconazole was obtained from Spectrum Chemicals (New Brunswick, NJ). AG1024, 3-nitroproprionic acid, pertussis toxin, and all other chemicals were obtained from Sigma (Saint Louis, MO), unless otherwise noted. Purified endotoxin-free recombinant HIV Tat (1–72) was made and characterized as previously described [9, 10]. Gp120 derived from HIV_IIIB_ and HIV_SF_ was acquired from the NIH-AIDS repository (Bethesda, MD, USA). HIV-gp120 transgenic mice were kindly provided by Dr. Mucke at the Gladstone Institute.

### Neuronal Cultures

Primary neuronal hippocampal cell cultures were established from E16-18 Sprague–Dawley rats. Cerebral hippocampi were dissected free of meninges, washed in Dulbecco’s phosphate-buffered saline (PBS), and digested using papain at 37 °C for 20 min. After trituration, cells were seeded at 4 × 10^4^ cells/well (96-well) and 7 × 10^5^ cells/well (6-well) on polyethylenimine-coated plates. Cells were maintained in neurobasal media supplemented with 2% (v/v) B-27 supplement, 1 mM HEPES, 2 mM glutamate, 5% (v/v) heat-inactivated fetal bovine serum (FBS), and 1% (v/v) antibiotic and antimycotic solution. Experiments were performed 12–15 days after plating.

Human neuronal cultures were prepared as described previously [11]. Briefly, the cells were mechanically dissociated; suspended in Opti-MEM with 5% (v/v) heat-inactivated FBS, 0.2% (v/v) N2 supplement, and 1% (v/v) antibiotic and antimycotic solution; and plated in flat-bottomed polyethylenimine-coated vessels. Neurons were plated at a density of 2 × 10^5^ cells/mL (15-mm diameter glass coverslips) or 4 × 10^5^ cells/mL (35-mm diameter dishes) and maintained for at least 1 month prior to experimentation.

Human neural progenitor cells (NPCs) were derived as described previously [12] and cultured in StemPro® NSC SFM–Serum-Free Human Neural Stem Cell Culture Medium (Thermo Fisher Scientific).

### Neuronal Progenitor Cell Proliferation

NPCs were plated into 96-well plates and cultured as described above. When cells were 60% confluent, they were treated with fluconazole (5 and 10 μM) for 8 h followed by 10 μM of EdU (Thermo Fisher Scientific) for an additional 24 h. Cells were fixed with 4% (w/v) paraformaldehyde (Sigma) and EdU labeling was detected using Click-iT EdU Imaging Kits (Thermo Fisher Scientific) according to the manufacturer’s instructions; 4’,6-diamidino-2-phenylindole (DAPI) was used for nuclear staining. Images of predetermined fields from each treatment group were taken and cell proliferation was determined by calculating the ratio of EdU-positive cells and total DAPI-positive cells.

### Neurotoxicity and Compound Screening

Cells (rat mixed hippocampal cultures or human neuronal cultures) were treated with DMSO (vehicle on control wells) or treated with test compounds for 1 h at 37 °C prior to addition of the neurotoxic insult 3-NOPA (3 mM), N-methyl-D-aspartic acid (NMDA, 100 μM), oxidopamine (OHDA, 100 μM), HIV Tat (500 nM), or gp120 (300 pM) for 18 h or H_2_O_2_ (100 µM) for 2 h. After incubation, cell viability was assessed by 3-(4,5-dimethylthiazol-2-yl)-2,5-diphenyltetrazolium bromide (MTT) assay. Briefly, viable cells cleave MTT resulting in an accumulation of formazan crystals which results in a colorimetric shift detectable by spectrophotometry [13]. Readings were obtained on a multiwell scanning spectrophotometer (SpectraMAX M5e, Molecular Devices). The viability of cells is directly proportional to the level of the formazan product created.

Throughput functional screening assays were performed as previously described [4, 13]. Briefly, rat mixed hippocampal cultures were treated with 3-NOPA (3 mM), which generates 25–35% cytotoxicity. Prior to the addition of 3-NOPA, 0.1% DMSO (vehicle) and compounds included in the screening were incubated at 10 µM for 1 h in a humid chamber at 37 °C and 5% CO_2_. Throughput screenings were performed in triplicate or quadruplicate wells and replicated three times. Confirmatory assays, including dose responses, were completed with eight replicates per treatment group and were repeated independently three times.

### Treatment of Neuronal Cultures

Neurons were cultured as described above. To perform testing, media were replaced with Opti-MEM containing the appropriate amount of compound to be tested as described for individual experiments. Pretreatment with vehicle control (0.1% DMSO) and fluconazole (1–10 μM) was performed as indicated prior to the addition of 3-NOPA (3 mM) and cells were measured for toxicity, as previously described, or lysates were prepared for immunoblotting. Cells were treated with pertussis toxin (PTX, 100 ng/mL), LY294002 (50 μM), AG1024 (5 μM), or DMSO (vehicle) for 1 h and then supplemented with 1–10 μM fluconazole for 4 h at 37 °C in a humidified chamber with 5% CO_2_. Cell lysates were then prepared for immunoblot analysis.

### Animals, Treatments, and Immunostaining

Heterozygous gp120 transgenic mice on a C57BL/6 background (described previously in [14] and [15]) were housed in a standard facility. A minimum of five animals per group were used for each experimental condition. Eight- to nine-week-old male wild-type (WT) and gp120 transgenic mice (tg) were intraperitoneally (ip) administrated with saline vehicle or with fluconazole (20 mg/kg) once per day for 28 days. For labeling newly generated cells, bromodeoxyuridine (BrdU; 50 mg/kg, Sigma-Aldrich, #B5002) was injected daily for 7 days during the second week of the experiment. For labeling of proliferating NPCs, 5-ethynyl-2-deoxyuridine (EdU; 50 mg/kg, Thermo Fisher Scientific, #E10187) was administered 3 h prior to euthanasia on day 28.

Brains were processed as described previously [14]. Briefly, animals were perfused with saline followed by 4% (w/v) paraformaldehyde (PFA) overnight. Brains were then stabilized in a 30% (v/v) sucrose solution, cryoprotected, and 40-µm thick sections were cut on a sliding microtome (Leica Biosystems). Brain sections spanning the entire hippocampal dentate gyrus were used for quantifying cell proliferation by immunostaining. After washing and blocking sections, primary antibodies anti-BrdU (rat, 1:1000; Accurate, #OBT0030) and anti-NeuN (mouse, 1:250; Chemicon, #MAB377B) were applied overnight at 4 °C, washed, and fluorescent secondary antibodies were applied for 2 h at room temperature. EdU labeling was detected using Click-iT EdU Imaging Kits (Thermo Fisher Scientific, #C10339) according to the manufacturer’s instructions. Sections were counterstained with DAPI to label all nuclei, mounted on Superfrost Plus glass slides, and imaged on a Zeiss LSM 510 Meta multiphoton confocal system (Carl Zeiss Microimaging Inc.).

For 3-NOPA-induced toxicity and fluconazole-mediated protection, fluconazole (20 mg/mL) was administrated ip once a day for 10 consecutive days. Mice were injected with 3-NOPA twice daily in an incremental increase dose of 20 mg/kg (for 2 days), 40 mg/kg (for 2 days), and 60 mg/kg (for 1 day) for a total of 5 days. Mice were weighed daily. After euthanasia, brains were processed as described above and lesions were detected by staining fixed sections with 0.1% (w/v) cresyl violet (Abcam, #ab246816) for 5 min at room temperature. Slides were washed, dehydrated, mounted, and imaged. Sections were prepared as described above and stained for dopamine- and cAMP-regulated neuronal phosphoprotein (DARPP-32, CST, #2306) overnight at 4 °C, washed, and fluorescent secondary antibodies were applied for 2 h at room temperature. Sections were counterstained with DAPI, mounted on Superfrost Plus glass slides, imaged as described above, and quantified.

### Caspase-3 Immunofluorescence

Immunofluorescence was performed for active caspase-3 on mixed primary hippocampal cultures pretreated with fluconazole (10 μM) or vehicle (DMSO) for 1 h followed by addition of media or 3-NOPA (3 mM) for 18 h. Cells were fixed with 4% (w/v) paraformaldehyde, permeabilized, and non-specific immunoreactivity was blocked with 1% (w/v) bovine serum albumin (BSA). Primary antibody to active caspase-3 (CST, #9664) was incubated at room temperature for 1 h followed by Alexa-Fluor 488-conjugated goat anti-rabbit IgG (Thermo Fisher Scientific) for 30 min. DAPI was used to visualize the nuclei. Coverslips were mounted onto slides with mounting solution. Images were captured using a computer-controlled Nikon Eclipse E600 fluorescent microscope (Nashville, TN).

### Cyclic AMP Enzyme Immunoassay

Rat mixed hippocampal cultures (4 × 10^4^ cells/well) were incubated with vehicle (DMSO) or fluconazole (1–10 μM) for 1 h. Cells were lysed with 0.25% v/v solution of dodecyltrimethylammonium bromide in 0.05 M acetate buffer (pH 5.8) containing 0.02% w/v BSA. Intracellular cAMP levels were measured using cAMP enzyme immunoassay (Amersham Pharmacia Biotech, Pittsburg, PA) according to the manufacturer’s instructions.

### Immunoblotting

Cells were lysed with RIPA buffer (50 mM Tris–HCL, 5 mM EDTA, 150 mM NaCl, 1% (v/v) Nonidet P-40, 0.5% (w/v) sodium deoxycholate, 0.1% (w/v) SDS, 10 mM NaF, 5 mM EGTA, 10 mM sodium pyrophosphate, and 1 mM phenylmethylsulfonylfluoride, pH 8.0) containing both protease and phosphatase inhibitors (Roche Diagnostics, Indianapolis, IN). Total protein was quantified by bicinchoninic acid (BCA) assay (Thermo Fisher Scientific, Rockford, IL). Protein extracts were resolved by sodium dodecyl sulfate (SDS)–polyacrylamide gel electrophoresis and transferred to nitrocellulose membranes. Immobilized proteins were analyzed by immunoblotting with 5% (w/v) non-fat milk and 0.1% (v/v) Tween-20 in PBS using the following CST antibodies: IGF-1/IR (#9750), HSP90 (#4874), panAkt (#9272), phos-Akt-T308 (#2965), and phos-Akt-S473 (#4060). Primary antibody incubations were performed overnight at 4 °C. Horseradish peroxidase (HRP) secondary antibodies (GE Healthcare, Pittsburgh, PA) were incubated at room temperature for 45 min. Membranes were developed with enhanced chemiluminescence (ECL, Amersham Pharmacia Biotech). After film development, membranes were stripped using Re-blot Plus stripping solution (Millipore, Billerica, MA) and re-incubated with primary antibodies as described above. Densitometric analyses of protein bands were performed using ImageJ software (National Institutes of Health, Bethesda, MD).

### Fluorescence Axodendritic Degeneration Assays

Rat mixed hippocampal neurons were transfected with a plasmid containing the neuron-specific CaMKII promoter, with a fusion construct of βIII tubulin-eGFP genes (pLV-CaMKII-βIII Tubulin-eGFP) with Nucleofector 2S (Lonza), according to the manufacturer’s instructions. Transfection efficiency was determined to be 60% by monitoring fluorescence. After 12–15 days in culture, cells were utilized in neurotoxicity assays to assess the effects of fluconazole on 3-NOPA-mediated axodendritic degeneration. Following 24 h of fluconazole (0.1–1 μM) and 3-NOPA (5 mM) treatment, live cell images were captured on a Zeiss AxioObserver Z1 inverted microscope or GE INCell Analyzer 2000 bioimager and the neurons expressing GFP were quantitated with GE Investigator analysis software as described previously [16].

### Statistical Analysis

All data are represented as mean ± standard deviation (SD) of the mean. Differences in 3-NOPA-induced in vitro toxicity were compared using an analysis of variance (ANOVA) with a Kruskal–Wallis correction for multiple comparisons. Differences in 3-NOPA-induced lesion volumes were compared using a two-tailed *t* test. Differences in body weight were compared by repeat measures mixed-effects model with a Holm-Sidak correction for multiple comparisons. Differences in 3-NOPA-mediated in vivo toxicity of DARPP32-positive neurons were compared by ANOVA with a Tukey’s correction for multiple comparisons. Differences in the percent of BrdU-positive cells normalized to DAPI were compared by ANOVA with a Dunnett’s correction for multiple comparisons. Differences in the percent of EdU- or BrdU- and NeuN-positive cells were compared by two-tailed *t* test. Differences in caspase-3 activation and neurite lengths were compared by ANOVA with a Tukey’s correction for multiple comparisons. Differences in cAMP levels were assessed by ANOVA with a Dunnett’s correction for multiple comparisons. Differences in optical densities (OD) of immunoblotting experiments were compared by ANOVA with either a Dunnett’s correction or a Holm-Sidak for multiple comparisons as described in the figure legends. *P* < 0.05 was considered statistically significant. All statistical analyses were performed with GraphPad Software 6.01 or SAS version 9.3.

## Results

### Identification of Azole Antifungal Agents as Neuroprotectants

**Fig. 1:**
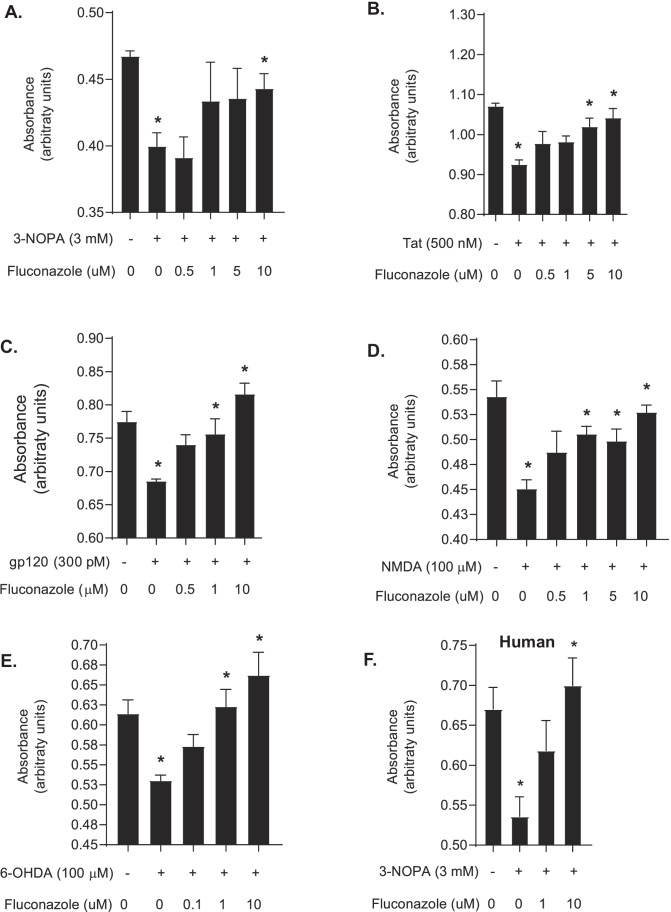
Fluconazole is protective against neurotoxic insults in vitro. **A**–**E** Bar graphs comparing cell viability as measured by MTT assay (absorbance, arbitrary units) of rat mixed hippocampal cultures preincubated with fluconazole (0–10 uM) for 1 h prior to exposure to toxic insult for 18 h. **A** Compared to control cells, 3-NOPA (3 mM) induced significant toxicity (*P* = 0.0006) which fluconazole (10 μM) prevented (*P* = 0.03). **B** Compared to control cells, Tat (500 nM) induced neurotoxicity (*P* < 0.0001) which fluconazole prevented at both 5 μM (*P* = 0.007) and 10 μM concentrations (*P* = 0.001). **C** Compared to control cells, HIV gp120 (300 pM) induced neurotoxicity (*P* = 0.006) which fluconazole prevented at 1 μM (*P* = 0.02) and 10 μM concentrations (*P* < 0.0001). **D** Compared to control cells, NMDA (100 μM) induced neurotoxicity (*P* < 0.001) which fluconazole could prevent at 1 μM (*P* = 0.006), 5 μM (*P* = 0.02), and 10 μM concentrations (*P* < 0.0001). **E** Compared to control cells, 6-OHDA (100 μM) induced neurotoxicity (*P* = 0.002) which fluconazole prevented at 1 μM (*P* = 0.002) and 10 μM concentrations (*P* = 0.0001). **F** Human fetal neurons (Human) were treated with 3-NOPA (3 mM) which resulted in neurotoxicity (*P* = 0.01) as compared to control cells which fluconazole (10 mM) prevented (*P* = 0.005). Statistical significance was determined by ANOVA, followed by Kruskal–Wallis test for multiple comparisons, *P < 0.05

Two thousand compounds (see [17]) were assessed for neuroprotective capacity against 3-nitropropionic acid (3-NOPA), which causes oxidative stress resulting in 25–35% cell death [4, 18, 19]. Each compound was tested at a single dose (10 µM). Several classes of drugs (Supplementary Table 1) showed neuroprotection from 3-NOPA-induced toxicity. Most notably, azole antifungal compounds including fluconazole (mean viability ± SD = 63.37 ± 14.99, *P* = 0.011), econazole (mean viability ± SD = 141.20 ± 34.14, *P* < 0.0001), itraconazole (mean viability ± SD = 87.50 ± 9.40, *P* = 0.0005), and miconazole (mean viability ± SD = 72.20 ± 9.15, *P* = 0.0035) demonstrated neuroprotection from 3-NOPA-induced toxicity (Table 1). Although fluconazole did not have the greatest level of neuroprotection, compared to other antifungal compounds, it has high bioavailability, including in the CNS, and an excellent safety profile (reviewed in [20]). Furthermore, fluconazole demonstrated the broadest neuroprotective profile with dose-dependent efficacy (Fig. 1). Fluconazole protected rat hippocampal neurons from 3-NOPA-induced damage, with an EC_50_ of 1–5 μM and maximal protection at 10 μM (Fig. 1A). Fluconazole also effectively protected neurons in a dose-dependent manner from other neurotoxins including HIV-1 Tat (Fig. 1B), HIV-1 gp120 (Fig. 1C), NMDA (Fig. 1D), and 6-hydroxydopamine (6-OHDA) (Fig. 1E). Utilizing human mixed neuronal cultures, the neuroprotective properties of fluconazole against 3-NOPA insult were confirmed. In this model, fluconazole demonstrated significant neuroprotection at 10 μM concentration (Fig. 1F), well within the typical bioavailable concentration of fluconazole taken orally. Based on the desirable safety and bioavailability of fluconazole, as well as our data showing broad neuroprotective capacity, we therefore selected fluconazole for further investigations.

**Table 1:** Antifungal agents are protective against oxidative stress induced by 3-NOPA

Compound name	Viability (%) normalized to 3-NOPA (mean ± SD)	*P* value*
Ketoconazole	20.93 ± 8.49	NS
Clotrimazole	66.50 ± 4.76	0.009
Fluconazole	63.37 ± 14.99	0.011
Sulconazole	18.23 ± 6.66	NS
Flutrimazole	46.50 ± 18.93	NS
Econazole	141.20 ± 34.14	< 0.0001
Miconazole	81.47 ± 49.58	0.001
Itraconazole	87.50 ± 9.40	0.005
Voriconazole	72.20 ± 9.15	0.004

^*^Data were analyzed by ANOVA with Dunnett’s correction

Abbreviations: *3-NOPA*, 3-nitropropionic acid; *NS*, not significant

### Fluconazole Is Neuroprotective In Vivo

**Fig. 2:**
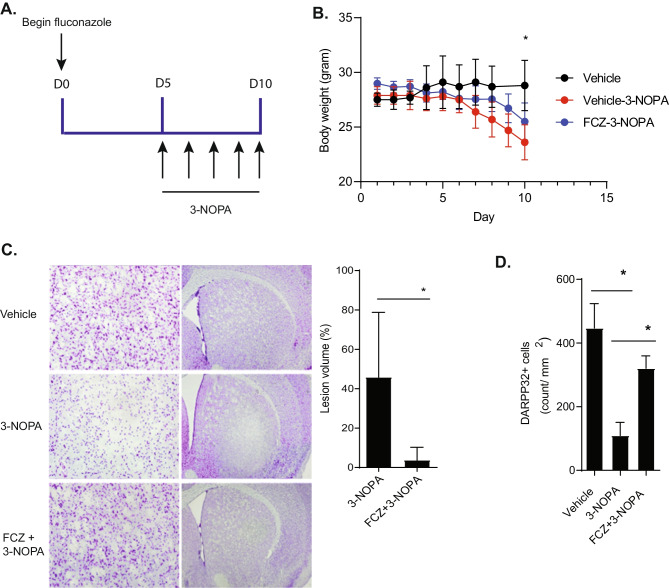
Fluconazole protects striatal neurons from 3-nitropropionic acid-mediated toxicity in vivo. **A** Experimental schema. Fluconazole was administrated intraperitoneally into animals with a dose of 20 mg/mL once a day for 10 consecutive days. Mice were injected with 3-nitropropionic acid (3-NOPA) twice daily in an incremental dose of 20 mg/kg for 2 days, 40 mg/kg for 2 days, and 60 mg/kg once, for a total of 5 days. **B** Body weight of vehicle-3-NOPA-treated (red) mice showed weight loss as compared to vehicle-only-treated mice (black) (*P* = 0.04). Mice treated with fluconazole prior to 3-NOPA exposure (FCZ/3-NP, blue) were protected from this weight loss (*P* = 0.002). Data were analyzed by repeat measures mixed-effects model with a Holm-Sidak correction for multiple comparisons, *P < 0.05. **C** Representative images (left) and quantification (right) of striatal lesion volume as shown by cresyl violet staining. As compared to 3-NOPA-treated mice, mice given fluconazole prior to 3-NOPA exposure showed higher cell density in the core of the lesion (*P* = 0.008). Statistical significance was determined by two-tailed *t* test, *P < 0.05. **D** Quantification of the number of striatal GABAergic medium spiny projection neurons expressing DAPRR32. Cell counts of DARPP32-positive neurons showed that 3-NOPA significantly decreased DARPP32-positive neurons in the lateral region of the striatum compared with control (*P* = 0.0005). Pretreatment with fluconazole prior to 3-NOPA exposure protected against this loss (*P* = 0.004). Statistical significance was determined by ANOVA, followed by Tukey’s test for multiple comparisons, *P < 0.05

To determine if fluconazole could protect neurons in vivo, mice were injected with 3-NOPA twice daily in escalating doses (20 to 60 mg/kg) for 5 days. Prior to 3-NOPA exposure, mice were treated with fluconazole (20 mg/mL) daily for 10 days (Fig. 2A). Body weight was monitored during treatment (Fig. 2B). Compared to vehicle-treated mice (mean body weight ± SD = 28.4 ± 0.7 g), mice treated with 3-NOPA lost weight (mean body weight ± SD = 26.7 ± 1.6 g, *P* = 0.04) which fluconazole prevented (mean ± SD = 27.8 ± 1.0 g, *P* = 0.002). There was no statistically significant difference in weight between vehicle-treated mice and mice pretreated with fluconazole prior to 3-NOPA exposure (*P* = 0.23). Injection of 3-NOPA induced toxicity which resulted in discrete lesions in the basal ganglia observable by cresyl violet staining (Fig. 2C). Quantification of lesion volume demonstrated that mice pretreated with fluconazole prior to exposure to 3-NOPA had a significant reduction in loss of neurons (percent mean lesion volume ± SD = 3.7 ± 6.5%, *P* = 0.008) as compared to 3-NOPA-treated mice (percent mean lesion volume ± SD = 45.9 ± 32.9%). Additionally, 3-NOPA resulted in significant loss of medium spiny neurons (Fig. 2D) as indicated by DARPP32 immunostaining. In mice treated with 3-NOPA, there was a reduction of DARPP32 positive cells (mean ± SD = 109.5 ± 117.0 cells per field,) as compared to vehicle-treated animals (mean ± SD = 447.0 ± 187.9 cells per field; *P* = 0.0008). Fluconazole pretreatment prevented this loss of DARPP32-positive cells (mean ± SD = 320.4 ± 146.9 cells per field; *P* = 0.01).

### Fluconazole Stimulates NPC Proliferation and Neuronal Generation In Vitro and In Vivo

**Fig. 3:**
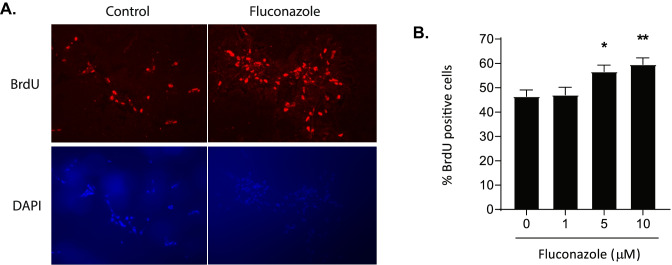
Fluconazole stimulates human neural progenitor cell proliferation in vitro. **A**–**B** Human neural progenitor cells (NPC) were treated with fluconazole (1–10 mM) for 8 h prior to adding bromodeoxyuridine (BrdU) to label the proliferating NPCs. After 24 h, the cells were fixed and stained for BrdU. **A** Representative photomicrographs of control- and fluconazole (10 mM)-treated cells. BrdU is shown in red, and DAPI, to label the nuclei, is shown in blue. **B** Quantification of the percent of BrdU-positive cells normalized to DAPI for each treatment group (*n* = 20 fields per group). Fluconazole significantly increased BrdU labeling in human NPCs at both 5 μM (*P* = 0.03) and 10 μM (*P* = 0.004) concentrations. Statistical significance was determined by ANOVA, followed by Dunnett’s correction for comparisons, *P < 0.05

To further understand the potential effects of fluconazole on tissue homeostasis and repair, we examined the impact of fluconazole on neural progenitor cells (NPCs). Human NPCs were treated with bromodeoxyuridine (BrdU), which is incorporated into dividing cells. Immunofluorescence staining for BrdU revealed that fluconazole increased NPC division in a dose-dependent manner (Fig. 3A, B). Fluconazole treatment at 5 µM (mean BrdU-positive cells (%) ± SD: 56.7% ± 11.3, *P* = 0.03) and 10 µM (59.5% ± 11.6, *P* = 0.004) showed increased BrdU-positive cells as compared to untreated cells (46.5% ± 11.2).

**Fig. 4:**
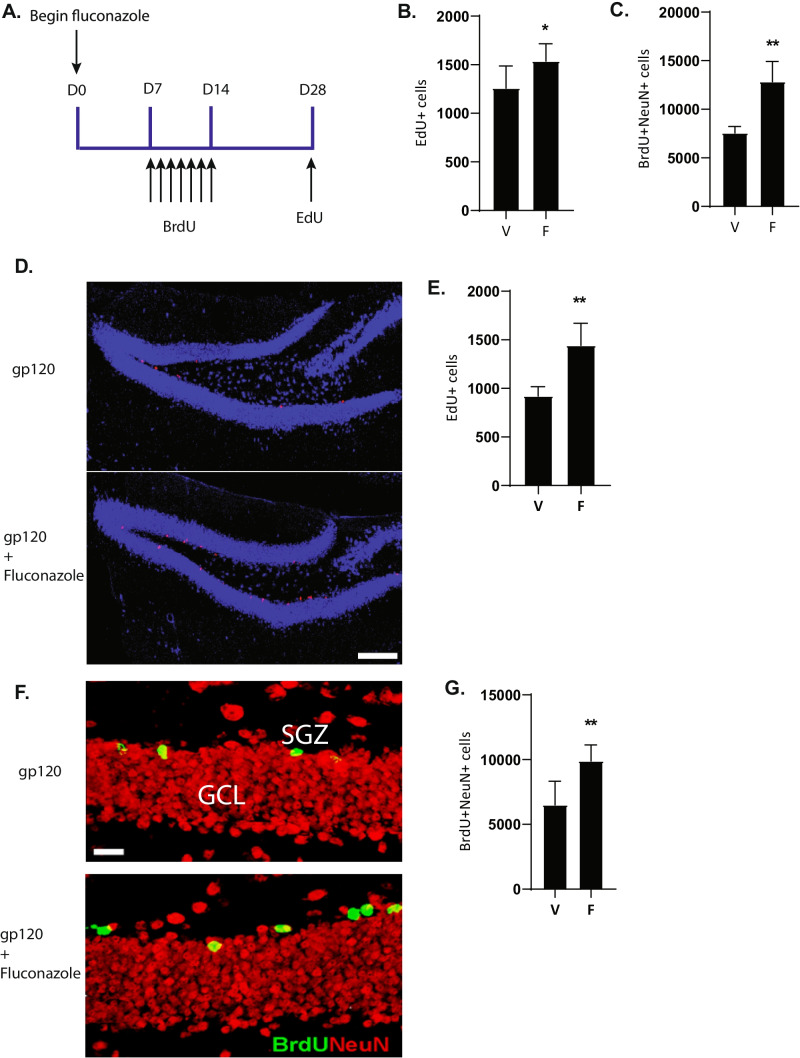
Fluconazole stimulates murine neural progenitor cell proliferation in vitro and in vivo. **A**–**C** Fluconazole stimulates proliferation of NPC in wild-type (WT) mice. **A** Experimental schema. Nine- to eight-week-old wild-type mice were intraperitoneally administrated with saline vehicle or with 20 mg/kg fluconazole once per day for 28 days. For labeling newly generated cells, bromodeoxyuridine (BrdU, 50 mg/kg) was injected daily for 7 days during the second week of the experiment. For labeling of proliferating NPCs, 5-ethynyl-2-deoxyuridine (EdU, 50 mg/kg) was administered 3 h prior to euthanasia on day 28. **B** Quantification of EdU-positive cells showed that fluconazole significantly increased NPC proliferation in WT mice as compared to vehicle-treated mice (*P* = 0.049; *V* = vehicle, *F* = fluconazole). **C** Quantification of newly formed neurons (dual positive for NeuN and BrdU) showed that fluconazole greatly increased the formation of new neurons (*P* = 0.0006). Statistical significance was determined by two-tailed t test, *P < 0.05. **D**–**G** Fluconazole rescues cell proliferation and adult hippocampal neurogenesis in gp120 transgenic mice. A similar experimental design as described above in (**A**) was performed on gp120 transgenic mice. **D** Photomicrographs of representative images of cells labeled with EdU (red) in the dentate gyrus (DG) of the hippocampus which identifies the newly generated cells (EDU +). **E** Quantification of the number of EdU-positive cells shows that fluconazole increases proliferating NPCs in gp120 transgenic mice (*P* = 0.0003) as compared to vehicle-treated mice. **F** Photomicrographs of representative images of cells double labeled with BrdU (green) and NeuN (red) which identifies the newly generated neurons (BrdU + NeuN +). SGZ, subgranular zone of the DG. GCL, granule cell layer of the DG. **G** Quantification of the number of dual positive BrdU and NeuN cells shows that fluconazole increases the number of newly generated cells that differentiate into neurons in gp120 transgenic mice as compared to vehicle-treated mice (*P* = 0.005). Statistical significance was determined by two-tailed *t* test, *P < 0.05

To determine if there was an effect on NPC proliferation and neurogenesis in vivo, WT mice were treated with fluconazole (20 mg/kg daily) for 28 days (Fig. 4A). Mice were then administered BrdU from day 7 through day 14 (50 mg/ kg daily) to label newly generated neurons, and on day 28, 3 h prior to euthanasia, mice were injected with 5-ethynyl-2-deoxyuridine (EdU) to label proliferating NPCs. Immunohistochemistry was performed on sections from the dentate gyrus to identify proliferating NPCs (Edu-positive cells) as well as newly generated neurons (BrdU- and NeuN-positive cells). Fluconazole treatment of WT mice resulted in a 20% increase in proliferation of NPCs (Fig. 4B) as compared to vehicle-treated WT mice (mean number of EdU-positive cells per mm^3^ ± SD: fluconazole treated, 1538 ± 177.7 versus vehicle treated, 1258 ± 229, *P* = 0.049). Furthermore, fluconazole increased the number of newly formed neurons (Fig. 4C). WT mice administered fluconazole had significantly more BrdU-/NeuN-positive cells as compared to vehicle-treated WT mice (mean number of BrdU-/NeuN-positive cells per mm^3^ ± SD: fluconazole treated, 12,843 ± 2080 versus vehicle treated, 7550 ± 669.1, *P* = 0.006).

To determine if fluconazole could rescue the loss of NPC proliferation due to a neurotoxin, gp120 transgenic (gp120 tg) mice were treated with fluconazole as described above (Fig. 4A). We observed an approximately 30% decrease in NPC proliferation in gp120 tg mice compared with WT mice, which was not significantly different from our previous study [21] (Supplemental Fig. 1). Fluconazole-treated gp120 tg mice had a significant increase in NPC proliferation (Fig. 4D) as compared to fluconazole-untreated gp120 tg mice (mean number of EdU-positive cells per mm^3^ ± SD: fluconazole treated, 1443 ± 228.4 versus vehicle treated, 920.5 ± 97.0, *P* = 0.0003) (Fig. 4E). Importantly, fluconazole also resulted in an increase in newly formed neurons in the gp120 tg model (Fig. 4F, G) as compared to untreated gp120 tg mice (mean number of BrdU-/NeuN-positive cells per mm^3^ ± SD: fluconazole treated, 9899 ± 1237 versus vehicle treated, 6503 ± 1818, *P* = 0.005). Collectively, these data demonstrate that fluconazole can stimulate NPC proliferation and neural development in vitro and in vivo, resulting in the formation of new neurons, even during a neurotoxic insult.

### Fluconazole Inhibits Caspase-3 Cleavage and Neurite Trimming In Vitro

**Fig. 5:**
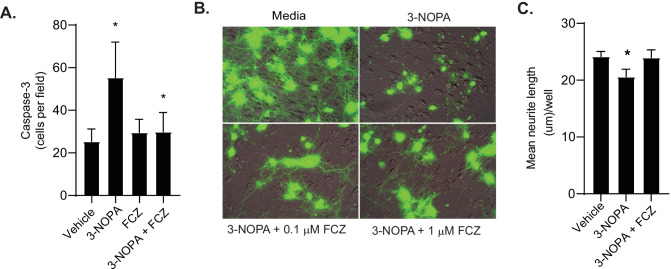
Fluconazole inhibits caspase-3 cleavage and neurite trimming in vitro. **A** Quantification of immunofluorescence for caspace-3 cleavage in rat mixed hippocampal cultures incubated with either vehicle or 10 μM fluconazole (FCZ) for 1 h prior to exposure to 3 mM 3-NOPA for 18 h. As compared to vehicle-treated cells, 3-NOPA increased caspase-3 cleavage (*P* < 0.001) which was mitigated by pretreatment with fluconazole (*P* < 0.001). **B** Representative photomicrographs of green fluorescent protein images visualized over phase contrast images of neuronal cultures expressing green fluorescent protein under control of the CaM kinase II promoter. Cells were treated with 3-NOPA (5 mM) for 24 h in the presence of vehicle (0.1% DMSO), 0.1 μM, or 1 μM fluconazole. After 24 h, cells were visualized. Control cultures show fine and long processes with multiple branch points, whereas 3-NOPA-treated cultures show shorter, stubby processes with irregular shape. Fluconazole-treated cultures also demonstrate longer and finer processes. **C** Quantification demonstrates that 3-NOPA causes a decrease in neurite length as compared to vehicle-treated cells (*P* = 0.0004), which pretreatment with fluconazole prevented (*P* = 0.0008). Data were analyzed by ANOVA with a Tukey’s correction for multiple comparisons, statistical significance, *P < 0.05

Neurotoxic insults often initiate apoptosis through activation of caspase-3. Therefore, we next determined if 3-NOPA induced caspase-3 activation in human fetal neurons in vitro. Quantification of immunofluorescence imaging revealed that 3-NOPA treatment increased caspase-3 activation (mean caspase-3 positive cells per field ± SD: 55.33 ± 16.7, *P* < 0.001) as compared to cells treated with vehicle (25.33 ± 5.9) (Fig. 5A). Fluconazole (10 μM) pretreatment for 1 h prior prevented active caspase-3 activation (29.89 ± 9.1, *P* < 0.001) when compared to 3-NOPA-treated cells.

As degeneration and fragmentation of axons and dendrites are common phenomena observed in vitro and in vivo prior to neurons dying (reviewed in [21]), we next assessed if fluconazole could protect cells from 3-NOPA-induced neurite degeneration. To visualize neuronal processes, cultures were transfected with a vector containing green fluorescent protein (GFP) under control of the CaM kinase II promoter. Visualization and quantification of neurite length were performed after neurons were left untreated or exposed to 3-NOPA for 24 h, with or without pretreatment with fluconazole (Fig. 5B). Untreated neurons in culture show fine and long processes with multiple branch points, whereas the 3-NOPA-treated cultures show short, stubby processes with irregular shape. Cells pretreated with fluconazole prior to 3-NOPA exposure showed long neuronal processes. Quantification of neurite length demonstrated that pretreatment with fluconazole could prevent 3-NOPA-induced neurite degeneration (Fig. 5C). Fluconazole-pretreated cells (mean neurite length/well ± SD: 23.95 µm/well ± 1.38) showed no significant difference in neurite length after 3-NOPA exposure as compared to untreated cells (24.16 µm/well ± 0.91, *P* = 0.96), whereas both fluconazole-pretreated (*P* = 0.004) and fluconazole-untreated cells (*P* = 0.002) had significantly longer neurites than 3-NOPA-treated cells (20.58 µm/well ± 1.37). These data indicate that fluconazole can prevent neurite degeneration and induction of apoptotic pathways in neurons.

### Fluconazole Decreases cAMP and Activates Akt

**Fig. 6:**
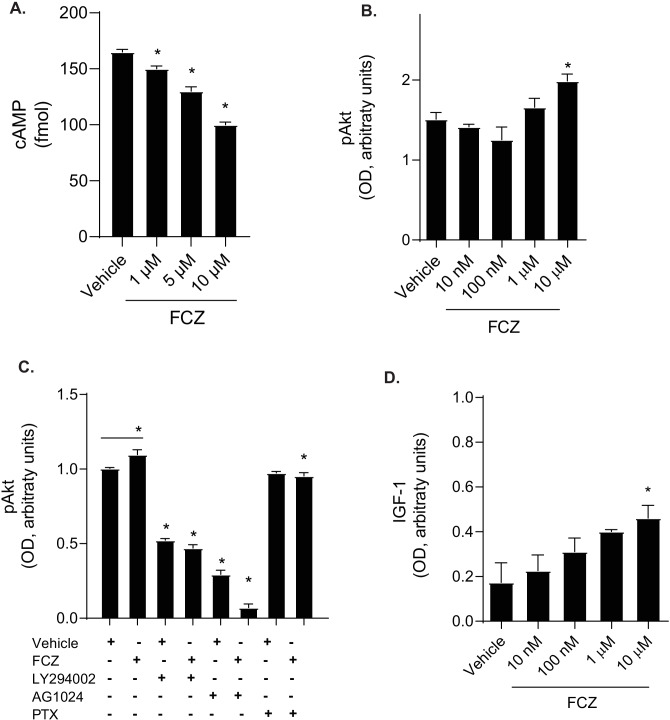
Fluconazole decreases cAMP, activates Akt, and drives expression of IGF-1 receptor. **A** Bar graph showing cAMP levels (fmol) from rat mixed hippocampal cultures incubated with vehicle or fluconazole (1–10 μM) for 1 h. Data were analyzed by ANOVA, followed by Dunnett’s correction for multiple comparisons. Statistical significance, *P < 0.05. **B** Bar graph showing phosphorylated Akt (pAkt) levels (optical density (OD), arbitrary units) as normalized to a loading control for rat neuronal cultures treated with vehicle or fluconazole (0.1–10 μM) for 4 h. Cells treated with 10 μM of fluconazole demonstrated a significant increase in Akt phosphorylation (*P* = 0.03). Statistical significance was determined by ANOVA, followed by Dunnett’s correction for multiple comparisons, *P < 0.05. **C** Bar graph showing phosphorylated Akt (pAkt) levels (OD, arbitrary units) as normalized to a loading control in rat neuronal cultures preincubated with compounds before the application of vehicle and fluconazole. Cells were pretreated for 1 h with the IGF-1 receptor inhibitor AG1024 (5 μM), the PI3K inhibitor LY294002 (50 μM), or the G-protein coupled receptor inhibitor pertussis toxin (100 ng/mL), followed with exposure to vehicle or fluconazole (1 μM) for 4 h. Statistical significance was determined by ANOVA, followed by a Holm-Sidak correction for multiple comparisons, *P < 0.05. **D** Bar graph depicting the levels of IGF-1 receptor (optical density, arbitrary units) as normalized to a loading control in rat neuronal cultures pretreated with AG1024 (5 μM) for 1 h followed by vehicle or fluconazole (0.1–10 μM) for 4 h. Statistical significance was determined by ANOVA, followed by a Dunnett’s correction for multiple comparisons, *P < 0.05

As neurite degeneration can lead to alterations and interruptions in neuronal communication, we next determined if fluconazole altered levels of the important secondary signaling molecule, cAMP. Neurons were exposed to either vehicle or increasing concentrations of fluconazole (1–10 μM) for 4 h. Cells were then washed, lysates harvested, and intracellular cAMP levels were measured via enzyme-linked immunosorbent assay (ELISA). Fluconazole decreased cAMP levels in a dose-dependent manner (Fig. 6A). Compared to untreated cells (mean cAMP ± SD = 165 ± 4.0), cells treated with fluconazole at 1 µM (150 ± 2.5, *P* = 0.0006), 5 µM (130 ± 3.9, *P* < 0.0001), or 10 µM (mean 100 ± 2.5, *P* < 0.0001) all showed decreases in cAMP levels. As cAMP is a negative regulator of Akt activation [22], and Akt signaling is important for neuronal survival (reviewed in [23]), we next investigated if fluconazole increased activation of Akt. Activation of Akt is accomplished via phosphorylation at threonine 308 and serine 473 (reviewed in [23]). Cells were left untreated or treated for 4 h with either vehicle or increasing amounts of fluconazole (10 nM–10 µM). Cells were harvested and cell lysates were prepared in lysis buffer with protease and phosphatase inhibitors. Immunoblots for Akt, phosphorylated (T308) Akt, and heat-shock protein 90 (HSP90) were performed. Treatments did not alter the levels of HSP90 indicating that global protein levels are not impacted by fluconazole. As such, levels of Akt and phosphorylated Akt were quantified and normalized to expression of HSP90. Compared to untreated cells (mean OD ± SD, 1.51 ± 0.08), 10 µM fluconazole (1.99 ± 0.09, *P* = 0.03) drove an increase in phosphorylated Akt (Fig. 6B, Supplemental Fig. 2A).

### Fluconazole Activates of Insulin-Like Growth Factor Receptor

Akt is an important downstream signaling molecule of phosphatidylinositol 3-kinase (PI3K). PI3K can be activated by multiple routes including receptor tyrosine kinases (RTKs) and G-protein coupled receptors (GPCRs). Growth factors such as epidermal growth factor (EGF), insulin-like growth factor 1 (IGF-1), and platelet-derived growth factor (PDGF) all signal through Akt activation (reviewed in [23]). To evaluate the mechanism by which fluconazole leads to Akt phosphorylation, we investigated the effect of inhibitors to GPCR, insulin-like growth factor 1 receptor (IGF-1R), and the PI3K pathway on fluconazole-mediated Akt phosphorylation.

Cells were either left untreated or preincubated with AG1024 (5 μM), an IGF-1 receptor inhibitor, LY294002 (50 μM), a PI3K inhibitor, or pertussis toxin (100 ng/ mL), a GPCR inhibitor, for 1 h. Cells were then exposed to fluconazole (1 µM) for 4 h, harvested, and analyzed for phosphorylated Akt by immunoblotting (Fig. 6C, Supplemental Fig. 2B). Fluconazole increased Akt phosphorylation (mean OD ± SD, 1.1 ± 0.05,) as compared to vehicle-treated cells (1.0 ± 0.01; *P* = 0.01). As expected, the PI3K inhibitor LY294002 decreased phosphorylation of Akt (0.52 ± 0.02, *P* < 0.001) as compared to vehicle-treated cells and was able to inhibit the induction of phosphorylation Akt by fluconazole (0.47 ± 0.04, *P* < 0.001). There was also a decrease in phosphorylated Akt expression in cells treated with AG1024 (0.30 ± 0.05, *P* < 0.001) as compared with vehicle-treated cells. AG1024 also robustly prevented the increase in Akt phosphorylation induced by fluconazole (0.07 ± 0.04, *P* < 0.001). Pertussis toxin had no impact on levels of Akt phosphorylation (0.97 ± 0.02, *P* = 0.31) as compared to vehicle-treated cells; however, it was capable of inhibiting fluconazole-induced Akt phosphorylation (0.95 ± 0.04; *P* = 0.005). These findings suggested that both IGF-1R, PI3K, and GPCRs may each mediate the induction of Akt phosphorylation by fluconazole.

### Fluconazole Increases Expression of IGF-1 Receptor

As AG1024, the IGF-1 receptor inhibitor, demonstrated a strong ability to block fluconazole-mediated phosphorylation of Akt, we next sought to determine if fluconazole had a direct effect on the IGF-1 receptor. To examine this, cells were treated with AG1024 (5 μM) for 1 h and then exposed to fluconazole for 4 h. Cells were harvested and IGF-1 receptor expression was analyzed by immunoblotting (Fig. 6D). Fluconazole increased IGF-1 receptor expression in a dose-dependent manner with significant upregulation at 10 μM (mean OD ± SD, 0.46 ± 0.06, *P* = 0.03) as compared vehicle-treated cells (0.17 ± 0.09). This increase of IGF-1 receptor and downstream activation of the Akt pathway may be the mechanism by which fluconazole mediates its neuroprotective effects.

## Discussion

The treatment of neurodegenerative diseases remains clinically challenging. Although much has been learned about the pathophysiological mechanisms of these diseases, no preventions or cures exist. In this study, we investigated the neuroprotective potential of 2000 compounds in an in vitro model of neurotoxicity with the ultimate goal of developing a broadly applicable pharmacological therapy for neuroprotection that could be safely taken for extended times. Our screening revealed that imidazole analogs, including antifungals and the selective serotonin reuptake inhibitor fluoxetine, have neuroprotective properties. We further investigated the effects of imidazoles on neurons in vitro and in vivo, using fluconazole as a candidate compound. We found that fluconazole was broadly neuroprotective in a dose-dependent manner against several classes of neurotoxic insults including oxidative stress, viral proteins, neurotoxins, and excitotoxicity. As compared to controls, fluconazole prevented neuronal death, inhibited neurite trimming, and blocked caspase-3 activation in vitro. In a murine model, fluconazole prevented neuronal death and protected against weight loss due to neurotoxin exposure and oxidative stress.

Although fluconazole did not demonstrate the greatest in vitro neuroprotective efficacy of the imidazoles examined, we selected it for further study due to its significant CNS penetration and excellent safety profile. Fluconazole, a triazole with an empirical formula of C_13_H_12_F_2_N_6_O, was approved by the FDA in 1990 as an antifungal agent. Fluconazole tightly binds to C-14-α-demethylases, thereby preventing the demethylation of the sterol at C14 and inhibiting ergosterol biosynthesis, leading to a weakening of the fungal cell wall [24]. Fluconazole is composed of two triazole rings and a difluorophenyl ring, and has a molecular mass of 306.27 Da, which is relatively small, as compared to other imidazole compounds. This low molecular weight facilitates CNS penetration and fluconazole achieves significant brain concentrations in rodents, non-human primates, and humans [25] with a half-life in the CNS of 27 h [26]. For example, patients with fungal meningitis exhibit fluconazole levels in the CSF at approximately 80% of the corresponding plasma concentrations regardless of the level of meningeal inflammation [27].

The neuroprotective effects exhibited by fluconazole against multiple forms of neuronal insults suggest a mechanism of action that impacts early signaling events. Fluconazole’s antifungal activities are linked to decreased levels of cAMP [28], which in *C. albicans* is mediated by the activation of phosphodiesterase Pde2 [29]. This enzyme catalyzes the conversion of cAMP to AMP. Indeed, imidazoles have been shown to activate several phosphodiesterases [30, 31], which in turn regulate the production of cAMP. Consistent with published observations, we found that fluconazole did cause a decrease in cAMP, a critical secondary signaling molecule in neurons. Since cAMP inhibits Akt (protein kinase B) activity [22], we next determined if fluconazole exposure led to increased Akt activation. We found that fluconazole induces phosphorylation of the threonine residue at position 308 of Akt, which is critical for its activation [23]. Importantly, Akt activation is known to inhibit apoptosis and strengthens pro-survival signaling, including in neurons [32, 33]. Collectively, our data suggest that fluconazole results in a decrease in cAMP, which drives an increase in Akt activation, resulting in decreased apoptosis in the face of neuronal insults.

Adenyl cyclase synthesizes cAMP in neurons from ATP [34]. IGF-1 activates the IGF-1 receptor and also triggers phosphorylation of β2-adrenergic receptor and inhibition of adenylyl cyclase, resulting in decreases in cAMP [35–37]. Furthermore, signaling through the IGF-1 receptor activates the Akt pathway and protects cells from apoptosis [38, 39]. The neuroprotective benefits of IGF-1 receptor ligation have been documented in models of excitotoxicity [40] and trauma [41] and IGF-1 plays a pivotal role in neuronal proliferation, differentiation, and migration [42–45]. As our studies indicated that fluconazole prevented apoptosis, drove Akt activation, and lowered cAMP levels, we next examined if these effects were mediated through IGF-1 receptor signaling. We found that the protective effects of fluconazole could be inhibited by AG1024, an IGF-1 receptor antagonist [46]. Furthermore, we demonstrated that fluconazole induced an increased expression of the IGF-1 receptor, although the mechanism underlying this upregulation remains unclear. Collectively, these data suggest that fluconazole exerts its neuroprotective effect by signaling through the IGF-1 receptor.

The IGF-1 receptor also drives neurogenesis by promoting proliferation and differentiation of neural progenitor cells [44, 45], and thus, this pathway is of great interest as a neurotherapeutic target. Ligation of the IGF-1 receptor by synthetic IGF-1 is used as a treatment for growth hormone insensitivity syndrome [47] and has recently been explored as the therapeutic for neurologic diseases such as myotonic dystrophy type 1 [48], Duchenne muscular dystrophy [49], and ALS [50]. In a clinical trial, patients with Duchenne muscular dystrophy given synthetic IGF-1 for 6 months demonstrated enhanced growth as compared to non-IGF-1-treated patients but did not exhibit improved walking distance [49]. Similarly, patients with ALS that received IGF-1 for 2 years did not demonstrate any significant difference as compared to untreated patients [50]. As fluconazole also signaled through the IGF-1 receptor, we investigated if fluconazole could promote neurogenesis. In both in vitro and in vivo, fluconazole stimulated the proliferation of neural progenitor cells and the formation of new neurons. These findings may explain the efficacy of fluconazole, in combination with paroxetine, in the prevention of neurodegeneration in macaques infected with simian immunodeficiency virus (SIV) [51]. Similar results were not observed in patients with HIV [52]. The lack of observable effect in this cohort may be due to the small number of patients in each treatment group (*n* = 9–11 people per group) as well as the short time (24 weeks) that patients were followed. It may be that with longer treatments, patients would have demonstrated improvement in the cognitive domains tested. Importantly, and different than the macaque model which treated animals 12 days after infection, the patients enrolled in this trial had long-standing HIV infections as well as substantial preexisting cognitive impairments. In our experiments, fluconazole was excellent at preventing neurodegeneration in vitro and in vivo, suggesting its clinical utility as a neuroprotective agent, but not necessarily at treating established cognitive impairment. The efficacy of fluconazole of preventing further clinical deterioration in patients with HIV-associated neurocognitive impairment or other neurodegenerative disorders has not been examined. However, as fluconazole may be an agonist of the IGF-1 receptor and may also drive the increased expression of this receptor, the possibility of targeting the IGF-1 receptor with this FDA-approved drug should be further investigated.

Our study has important limitations when considering the translational implications of this work. Fluconazole is known to be metabolized by cytochrome P450 enzymes and chronic treatments with fluconazole can alter expression and enzymatic activity of cytochrome P450 [53–55]. As cytochrome P450 is an important factor in drug pharmacokinetics [56], chronic treatment with fluconazole could alter cellular metabolism or metabolism of other drugs including antiretroviral therapies, blood thinners, and statins [53, 57, 58]. As understanding these interactions would be critical prior to the utilization of fluconazole as a neuroprotectant in diseases like HIV-associated neurocognitive impairments, future studies to examine the effects of chronic use of fluconazole on metabolism and drug interactions in disease-specific models are warranted. In conclusion, we demonstrated that fluconazole was broadly neuroprotective and stimulated neurogenesis in vitro and in vivo. Furthermore, we show that IGF-1 receptor signaling and downstream cAMP decreases and Akt activation are an important mechanism underlying these effects of fluconazole. Further studies into the utility and safety of fluconazole as a neuroprotectant during neurodegenerative disease are warranted.

## Supplementary Information

Below is the link to the electronic supplementary material.

Supplementary file1 (PDF 542 KB)

Supplementary file2 (DOCX 13 KB)

Supplementary file3 (PDF 516 KB)

Supplementary file4 (PDF 516 KB)

Supplementary file5 (PDF 516 KB)

Supplementary file6 (PDF 516 KB)

Supplementary file7 (PDF 524 KB)

Supplementary file8 (PDF 516 KB)

Supplementary file9 (PDF 1038 KB)

Supplementary file10 (PDF 516 KB)

Supplementary file11 (PDF 516 KB)

## Data Availability

Disclosure forms provided by the authors are available with the online version of this article.
