# Supplementary figures and images for: Fluconazole Is Neuroprotective via Interactions with the IGF-1 Receptor
Source: Neurotherapeutics. 2022 Jul 13;19(4):1313–28. doi: 10.1007/s13311-022-01265-0 (PMC9587198; doi:10.1007/s13311-022-01265-0)

S1.

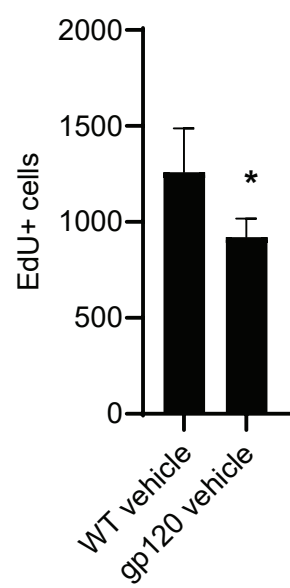

S2.

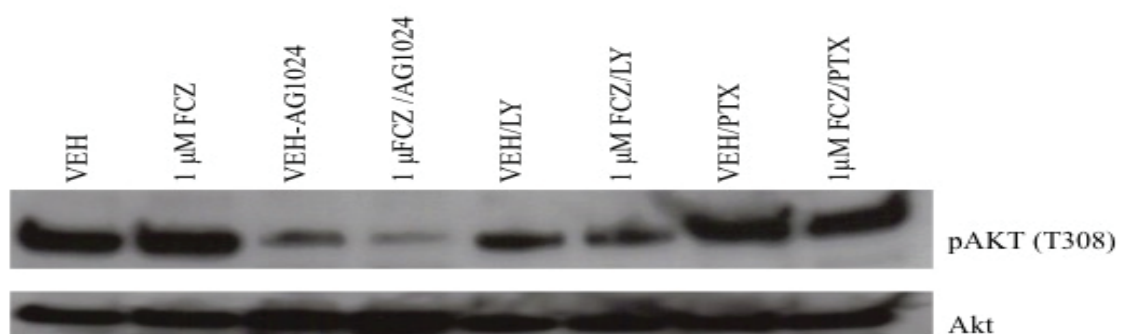

Supplement: Supplementary file 1 — Supplementary file1 (PDF 542 KB) [file 13311_2022_1265_MOESM1_ESM.pdf]
