# Supplementary material for: Fluconazole Is Neuroprotective via Interactions with the IGF-1 Receptor
Source: Neurotherapeutics. 2022 Jul 13;19(4):1313–28. doi: 10.1007/s13311-022-01265-0 (PMC9587198; doi:10.1007/s13311-022-01265-0)
Supplement: Supplementary file 2 — Supplementary file2 (DOCX 13 KB) [file 13311_2022_1265_MOESM2_ESM.docx]

Table S1. Other compound classes and example compounds demonstrating protective effects against oxidative stress induced by 3-NOPA.

| Compound class | Compound name | Viability (%) normalized to 3-NOPA  (Mean) |
| --- | --- | --- |
| SSRI | Fluoxetine | 101 |
|  | Paroxetine | 56 |
| Tricyclic antidepressant | Desipramine | 42 |
|  | Trimipramine | 95 |
|  | Amitriptyline | 44 |
|  | Nortriptyline | 143 |
| Flavanol | epicatechin | 50 |
|  | catechin | 42 |
| Estrogen ligands | Dienestrol | 83 |
|  | Estriol ester | 58 |

Abbreviations: 3-NOPA, 3-nitropropionic acid
